# Improvement of Platelet Respiration by Cell–Permeable Succinate in Diabetic Patients Treated with Statins

**DOI:** 10.3390/life11040288

**Published:** 2021-03-28

**Authors:** Vlad Florian Avram, Anca Mihaela Bîna, Alexandra Sima, Oana Maria Aburel, Adrian Sturza, Ovidiu Burlacu, Romulus Zorin Timar, Danina Mirela Muntean, Eskil Elmér, Octavian Marius Crețu

**Affiliations:** 1 Department VII Internal Medicine—Diabetes, Nutrition and Metabolic Diseases, “Victor Babeș” University of Medicine and Pharmacy, Eftimie Murgu Sq. no. 2, 300041 Timișoara, Romania; avram.vlad@umft.ro (V.F.A.); sima.alexandra@umft.ro (A.S.); timar.romulus@umft.ro (R.Z.T.); 2 Center for Translational Research and Systems Medicine, ”Victor Babeș” University of Medicine and Pharmacy, Eftimie Murgu Sq. no. 2, 300041 Timișoara, Romania; lungu.anca@umft.ro (A.M.B.); oanaduicu@umft.ro (O.M.A.); sturza.adrian@umft.ro (A.S.); 3 Department III Functional Sciences—Pathophysiology, “Victor Babeș” University of Medicine and Pharmacy, Eftimie Murgu Sq. no. 2, 300041 Timișoara, Romania; 4 Department IX Surgery—Surgical Semiotics I, “Victor Babeș” University of Medicine and Pharmacy, Eftimie Murgu Sq. no. 2, 300041 Timișoara, Romania; burlacu.ovidiu@umft.ro (O.B.); octavian.cretu@umft.ro (O.M.C.); 5 Center for Hepato–Biliary and Pancreatic Surgery, “Victor Babeș” University of Medicine and Pharmacy, Eftimie Murgu Sq. no. 2, 300041 Timișoara, Romania; 6 Mitochondrial Medicine, Department of Clinical Sciences, Faculty of Medicine, Lund University, BMC A13, 221 84 Lund, Sweden; 7 Abliva AB, Medicon Village, 223 81 Lund, Sweden

**Keywords:** platelets, diabetes, statins, cell–permeable succinate (NV118), mitochondria, high–resolution respirometry

## Abstract

Diabetes mellitus (DM) is the most severe metabolic disease that reached the level of a global pandemic and is associated with high cardiovascular morbidity. Statins are the first–line lipid–lowering therapy in diabetic patients with or without a history of atherosclerotic disease. Although well tolerated, chronic treatment may result in side effects that lead to treatment interruption. Mitochondrial dysfunction has emerged as a central pathomechanism in DM– and statin–induced side effects. Assessment of mitochondrial respiration in peripheral platelets has been increasingly used as a mirror of organ mitochondrial dysfunction. The present study aimed to assess the: (i) changes in mitochondrial respiration elicited by statins in patients with type 2 DM and (ii) the effects of cell–permeable succinate (NV118) on respiratory parameters in platelets harvested from these patients. No significant changes were found in global mitochondrial respiration of intact platelets isolated from diabetic patients treated with either atorvastatin or rosuvastatin. Similarly, no significant changes in mitochondrial respiration of permeabilized platelets were found between diabetic patients treated with atorvastatin and healthy controls. Acute ex vivo administration of NV118 significantly improved respiration in isolated platelets. These results prompt further research on the role of permeable succinate as a therapeutic alternative for improving mitochondrial function in metabolic pathologies and point to the role of peripheral platelets as a potential biomarker of treatment response.

## 1. Introduction

Atherosclerotic cardiovascular death is the leading cause of mortality worldwide, and statins are the first–choice agents in current guidelines for combating cardiometabolic disorders and reducing cardiovascular risk in both primary and secondary prevention [1,2]. Diabetes mellitus (DM) is the most severe metabolic disease, affecting almost half a billion people around the world [3] with increased prevalence in the pediatric population [4], and is commonly associated with obesity and dyslipidemia [5]. Currently, mitochondrial dysfunction has been proposed as a central pathomechanism of DM [6,7]. Grubelnik et al. recently demonstrated, in an elegant computational modeling approach, that impairment of both insulin and glucagon pancreatic secretion is linked to a reduced mitochondrial ATP generation [8].

Statins are the first–line lipid–lowering therapy in diabetic patients with or without a history of atherosclerotic disease. Although normally well tolerated, in some cases, statin treatment has been hindered by the occurrence of statin–induced muscle symptoms [9,10]. Mitochondrial dysfunction has also emerged as a central event in the pathophysiology of statin–induced myopathy [11,12,13,14], with mitochondrial complex I dysfunction being among the most cited mechanisms [13,15,16,17]. It has also been reported that mitochondrial respiration supported by complex I substrates is decreased in diabetic states [18]. Moreover, obesity seems to cause a similar dysfunction of complex I, leading to decreased oxidative phosphorylation [19]. This poses a dilemma when it comes to general mitochondrial health because diabetic patients are generally obese [3] and require statin treatment for associated dyslipidemia that increases cardiovascular risk and promotes chronic endothelial dysfunction [1,20]. The number of patients who discontinue statin therapy is alarming considering the prevalence of dyslipidemia and atherosclerotic cardiovascular disease across the world [2]. 

Cell–permeable succinates have been successfully used to rescue mitochondrial respiration in experimental models of acute complex I inhibition [21,22]. We recently showed in acute experiments using human platelets and HepG2 cells that a cell–permeable succinate prodrug, NV118, can bypass complex I mitochondrial dysfunction induced by statins [23]. 

In the past decade, peripheral platelets have been increasingly used as a source of viable mitochondria in order to investigate respiratory impairment as a mirror of organ–related mitochondrial dysfunction in various pathologies [24]. As mitochondrial dysfunction has been tied to both DM and statin treatment [9,10,15,16,17,18], we thought to perform an in–depth study of high–resolution respirometry in platelets harvested from diabetic patients chronically treated with statins.

Thus, the current study was aimed to assess whether: (i) chronic therapy with the most potent statins will impact platelet respiration and (ii) the ex vivo administration of NV118 will improve platelet mitochondrial bioenergetics in diabetic patients.

## 2. Materials and Methods

### 2.1. Study Population

Diabetic patients not treated (DM group) and treated with atorvastatin (DM + Atorvastatin group) and rosuvastatin (DM + Rosuvastatin group) were recruited from the Clinic of Diabetes, Nutrition and Metabolic Diseases of "Pius Brînzeu” County Emergency Hospital of Timișoara, Romania. The control group consisted of healthy volunteers. 

The study was performed in accordance with the tenets of the Declaration of Helsinki, and study protocols were approved by the Committee of Research Ethics of “Victor Babeș” University of Medicine and Pharmacy, Timisoara, Romania (No. 43/20.12.2018). Written informed consent was provided by all participants after the experimental procedures were explained. Demographic and laboratory data of participants are summarized in Table S1. Comorbidities and their related medication are listed in Table S2 (see Supplementary Materials).

### 2.2. Platelet Isolation

Venous blood from healthy volunteers and diabetic patients undergoing (or not) chronic treatment with statins was drawn in K_2_EDTA tubes. Platelets were isolated according to a previously described protocol [25] that uses 2 centrifugations. The first centrifugation at 500× *g* for 10 min resulted in platelet–rich plasma, whereas after the second centrifugation at 4600× *g* for 5–10 min, the platelet pellet was obtained and used for the assessment of respiratory function by high–resolution respirometry. Platelets were resuspended in their own plasma. Platelet count was assessed using a Sysmex hematology analyzer.

### 2.3. High–Resolution Respirometry (HRR)

Mitochondrial respiration was assessed with the O2k–Oxygraph (Oroboros Instruments GmbH Innsbruck, Austria) and a buffer (MiR05) containing: 0.5 mM EGTA, 3 mM MgCl_2_, 60 mM K–lactobionate, 20 mM taurine, 10 mM KH_2_PO_4_, 20 mM HEPES, 110 mM sucrose and 1 g/L bovine serum albumin [26]. Platelets were suspended in the 2 mL glass chamber at a concentration of 200 × 10^6^ cells/mL at 37 °C. All chemicals were obtained from Sigma–Aldrich. The cell–permeable succinate prodrug NV118 was kindly provided by Abliva AB (Lund, Sweden). It is also available via Oroboros Instruments in the MitoKit-CII (https://www.oroboros.at/index.php/product/mitokit-cii/ (accessed on 22 March 2021)).

### 2.4. Experimental Protocols

Platelet mitochondrial respiration was evaluated in two separate protocols:

**Protocol A (Intact Cells):** Platelets from diabetic patients treated with either atorvastatin (n = 4) or rosuvastatin (n = 4) and nontreated diabetic controls (n = 4) were harvested as previously described and evaluated by means of HRR. Platelet oxygen consumption was allowed to stabilize until reaching a steady state (ROUTINE respiration). Intact platelets were then exposed to either the cell–permeable succinate NV118 (500 µM) or an equivalent volume of DMSO (as control). ATP–synthase was inhibited using oligomycin (1 μg/mL) in order to assess mitochondrial respiration independent of the phosphorylation process (LEAK respiration), after which consecutive titrations of CCCP (a protonophore) were added to reveal the maximal activity of the electron transport system (ETS), i.e., the ET capacity. To further investigate the direct effects of NV118 on complex–II–supported respiration, complex I was inhibited with rotenone (2 µM). Finally, specific inhibition of complex III with antimycin A (1 µg/mL) allowed for the assessment of the residual oxygen consumption (ROX).

In order to further dissect the effects of chronic statin treatment on mitochondrial respiration, a separate set of experiments was carried out using permeabilized platelets from diabetic patients chronically treated with atorvastatin (n = 4) vs. nontreated diabetic controls (n = 4) according to Protocol B.

**Protocol B (Permeabilized Cells):** Platelets were harvested as previously described and evaluated by means of HRR. Platelet oxygen consumption was allowed to stabilize at ROUTINE respiration in MiR05 without exogenous substrates. Platelets were then permeabilized with digitonin (1 µg/L × 10^6^ platelets) in order to allow the mitochondrial access of respiratory substrates and ADP. Malate (5 mM), pyruvate (5 mM), ADP (1 mM) and glutamate (5 mM) were added to saturate the NADH–linked pathway, after which succinate (10 mM) was added to do the same for the succinate–linked pathway. The addition of oligomycin (1 µg/mL) allowed for the assessment of LEAK respiration (respiration independent of ADP phosphorylation). The protonophore CCCP was titrated to measure the maximal noncoupled respiration. In order to account for nonmitochondrial respiration, complex I was then inhibited by using rotenone (2 µM), and the same was done for complex III using antimycin A (1 µg/mL). Permeabilized data from the DM group were omitted due to unusual sensitivity to digitonin/storage.

**Figure 1:**
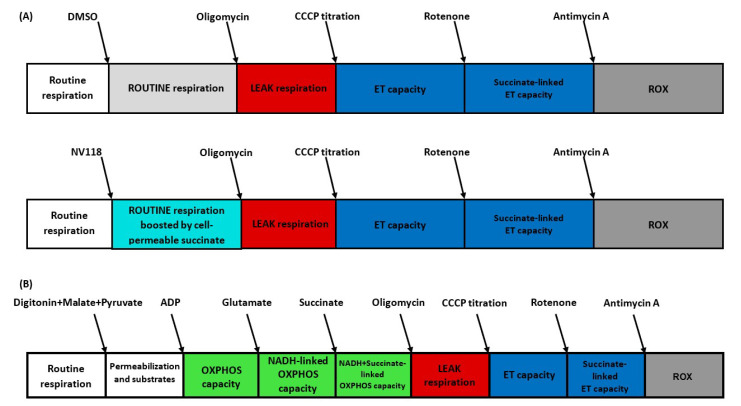
**Experimental protocols for the study of platelet mitochondrial respiration by HRR.** (**A**) Intact cell protocol. (**B**) Permeabilized cell protocol. ADP = adenosine diphosphate; CCCP = carbonyl cyanide m–chlorophenyl hydrazone; ET = electron transport; HRR = high–resolution respirometry; OXPHOS = oxidative phosphorylation; NADH = nicotinamide adenine dinucleotide reduced form; ROX = residual oxygen consumption.

The experimental protocols performed in intact (A) and permeabilized (B) platelets are depicted in Figure 1.

### 2.5. Respiratory Parameters

The following mitochondrial respiratory parameters adapted from [27,28] were evaluated:

For Protocol A:

- ROUTINE respiration: mitochondrial oxygen consumption in the physiological coupling state;
- LEAK respiration (nonphosphorylating respiration): mitochondrial oxygen consumption after inhibition of ATP–synthase, a dissipative component of mitochondrial respiration;
- ET capacity: mitochondrial oxygen consumption in a fully uncoupled state, achieved by the titration of optimum concentration of CCCP (protonophore);
- R–L net ROUTINE capacity: calculated by subtracting LEAK respiration from ROUTINE respiration;
- E–L net ET capacity: calculated by subtracting LEAK respiration from ET capacity;
- R–L control efficiency: calculated by subtracting LEAK respiration from ROUTINE respiration and then dividing the result by the ROUTINE respiration. It is a measure of the state of coupling (ATP generation) of the ETS;
- E–L coupling efficiency: calculated by subtracting LEAK respiration from ET capacity and then dividing the result by the ET capacity. It is a measure of the state of coupling (ATP generation) of the ETS;
- Residual succinate–supported respiration: mitochondrial oxygen consumption after the inhibition of complex I using rotenone.

For Protocol B:

- NADH–linked OXPHOS capacity: mitochondrial oxygen consumption at saturating concentrations of ADP and complex I substrates;
- OXPHOS capacity (phosphorylating respiration): mitochondrial oxygen consumption at saturating concentrations of ADP with both complex I and complex II substrates;
- LEAK respiration (nonphosphorylating respiration): mitochondrial oxygen consumption after inhibition of ATP–synthase, a dissipative component of mitochondrial respiration;
- ET capacity: mitochondrial oxygen consumption in a fully noncoupled state, achieved by the titration of optimum concentration of CCCP (protonophore);
- Succinate–linked ET capacity: mitochondrial oxygen consumption in a fully noncoupled state, achieved by the titration of optimum concentration of CCCP (protonophore) after the inhibition of complex I using rotenone;
- P–L control efficiency: calculated by subtracting LEAK respiration from OXPHOS capacity and then dividing the result by the OXPHOS capacity. It is a measure of the state of coupling (ATP generation) of the ETS;
- E–L coupling efficiency: calculated by subtracting LEAK respiration from ET capacity and then dividing the result by the ET capacity as a measure of the degree of coupling (ATP generation).

### 2.6. Data Analysis

Statistical analysis was performed using GraphPad software (GraphPad Software version 9.0). All data are expressed as the mean ± SEM. All data were corrected for nonmitochondrial oxygen consumption, to account for the presence of residual oxygen consumption according to [27]. A paired *t*–test, an unpaired *t*–test and one–way ANOVA with Bonferroni’s post hoc test were performed for antimycin–corrected data. 

## 3. Results

### 3.1. Mitochondrial Respiration Is Not Decreased in Intact Platelets of Diabetic Patients Treated with Statins

**Figure 2:**
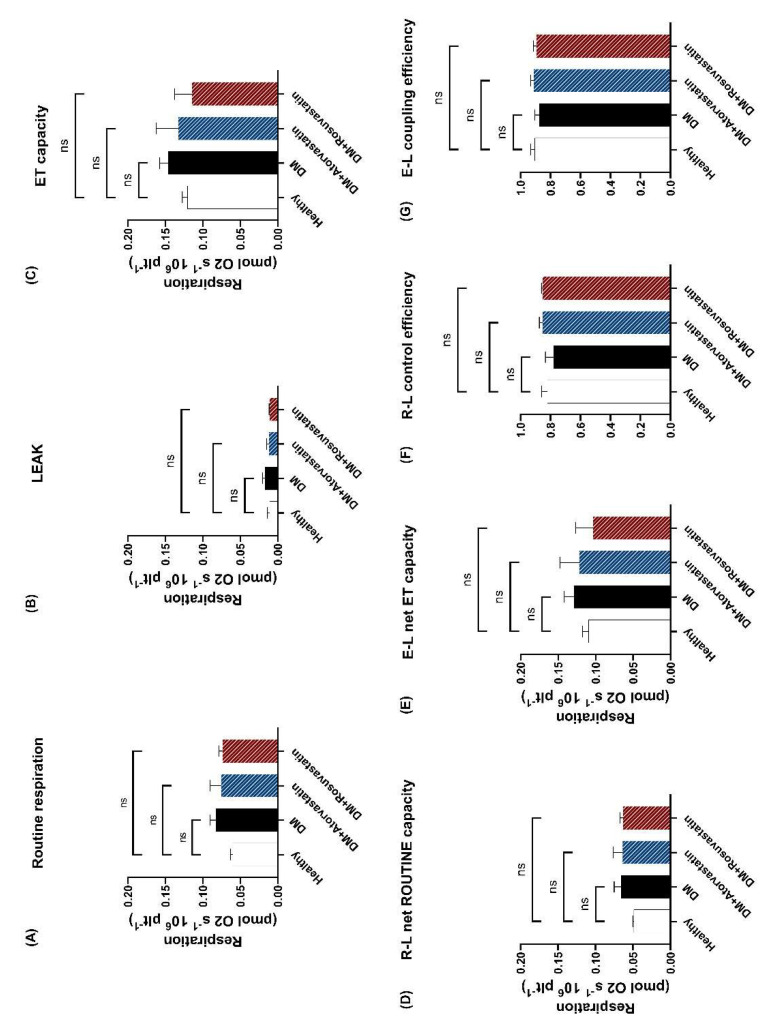
**Mitochondrial respiration in intact platelets isolated from diabetic patients treated with statins.** ROUTINE respiration (**A**), LEAK respiration (**B**), ET capacity (**C**) R–L net ROUTINE capacity (**D**), E–L net ET capacity (**E**), R–L control efficiency (**F**) and E–L coupling efficiency (**G**) were determined for three separate groups of diabetic patients (not treated with statins (black column), patients treated with atorvastatin (blue column) and patients treated with rosuvastatin (red column)) and compared to a group of healthy controls (white column). N = 4. Data are expressed as the mean ± SEM of antimycin A–corrected respiration. One–way ANOVA with Bonferroni’s post hoc test was performed. Ns = no statistical significance vs. control. E = electron transfer capacity, ET capacity = electron transfer capacity; L = LEAK respiration; R = ROUTINE respiration.

The first aim of the study was to assess the effects of chronic statin treatment on respiratory parameters in diabetic patients with no treatment (DM) compared to the treated groups (DM + Atorvastatin and DM + Rosuvastatin) and the control (healthy) patients, respectively. ROUTINE respiration (Figure 2A) was determined as oxygen consumption with endogenous substrates after the addition of DMSO and did not differ between statin–treated vs. nontreated patients. To exclude the possibility that mitochondrial respiration in the statin–treated patients might be nonphosphorylating and thus inefficient, we evaluated the LEAK respiration (non–ATP–generating respiration). Interestingly, a slight reduction (0.011 ± 0.003 in the DM + Atorvastatin group and 0.010 ± 0.001 in the DM + Rosuvastatin group) was found as compared to the statin–naïve patients (0.017 ± 0.003) that did not reach statistical significance (Figure 2B). The ET capacity that reflects the maximal activity of the ETS (Figure 2C) was evaluated as oxygen consumption in the presence of optimum concentrations of the protonophore CCCP and was comparable among the groups. To measure ATP–generating respiration, we calculated the R–L net ROUTINE capacity (Figure 2D), and no significance was found for the treated groups. The potential respiration available to the ETS to phosphorylate ADP (E–L net ET capacity) was further calculated (Figure 2E) and again was found unchanged by the statin treatment. These results suggest that the therapeutic doses of statins are safe in long–term administration for the mitochondria of diabetic patients at least when assessed at the platelet level. To further assess the efficiency of ATP–generation, we calculated the R–L control efficiency (Figure 2F) and the E–L coupling efficiency (Figure 2G) of the ETS. No statistical significance was found among the groups, suggesting that chronic statin treatment in diabetic patients does not affect phosphorylation efficiency. Moreover, in intact platelets, mitochondrial respiration parameters did not differ between diabetic patients and healthy controls.

### 3.2. Mitochondrial Respiration Is Not Decreased in Permeabilized Platelets of Diabetic Patients treated with Statins

In a separate set of experiments carried out in platelets harvested from diabetic patients treated with atorvastatin and the healthy control group, platelet membranes were permeabilized with digitonin to provide the access of exogenous oxidizable substrates and ADP and to separately assess the respiratory capacities through complex I and complex II. OXPHOS capacity was determined after the successive addition of specific substrates for both complex I and complex II along with ADP (in saturating concentrations).

**Figure 3:**
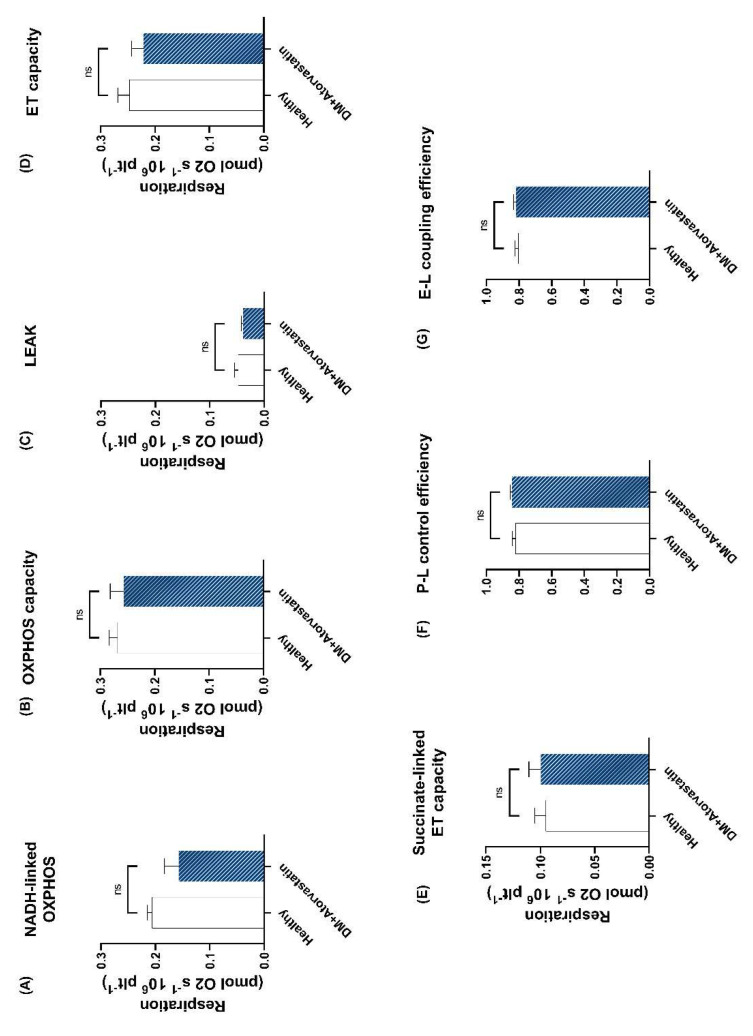
**Mitochondrial respiration in permeabilized platelets isolated from atorvastatin–treated diabetic patients.** (**A**) NADH–linked OXPHOS capacity was determined as oxygen consumption at saturating concentrations of ADP using only complex I substrates. (**B**) OXPHOS capacity was determined as mitochondrial oxygen consumption at saturating concentrations of ADP using both complex I and complex II substrates. (**C**) LEAK respiration was determined after the inhibition of ATP–synthase by oligomycin. (**D**) ET capacity was determined as oxygen consumption in an optimum state of uncoupling (reached through titration of CCCP). (**E**) Succinate–linked ET capacity determined in the fully noncoupled state, following the inhibition of complex I via rotenone addition. (**F**) P–L control efficiency and (**G**) E–L coupling efficiency were calculated as measures of mitochondrial ATP generation. N = 4. Data are expressed as the mean ± SEM of antimycin A–corrected respiration. Unpaired *t*–tests were performed. ns = no statistical significance vs. healthy control. E = electron transfer capacity; ET capacity = electron transfer capacity; L = LEAK respiration; OXPHOS = oxidative phosphorylation; P = oxidative phosphorylation; R = ROUTINE respiration.

In the permeabilized setting, it was possible to separately assess the NADH–linked OXPHOS, which presented a mild decrease (0.157 ± 0.025 vs. 0.206 ± 0.008) in the DM + Atorvastatin group vs. the healthy group (Figure 3A). No changes were found in the OXPHOS capacity (Figure 3B) of platelets isolated from diabetic patients treated with atorvastatin vs. controls. The nonphosphorylating (LEAK) respiration (Figure 3C) of permeabilized platelets was slightly lower in the DM + Atorvastatin group compared to the healthy group but with no statistical significance (0.039 ± 0.002 vs. 0.047 ± 0.006). No changes were found in the ET capacity (Figure 3D) of platelets isolated from diabetic patients treated with atorvastatin vs. controls. Succinate–linked ET capacity was also determined in the noncoupled state after the inhibition of complex I with rotenone and showed similar values between healthy individuals and the atorvastatin–treated diabetics (Figure 3E). To further assess the efficiency of ATP–generation, we calculated the P–L control efficiency (Figure 3F) and the E–L coupling efficiency (Figure 3G) of the ETS and found similar values between the two groups.

### 3.3. Cell Permeable Succinate Improved Mitochondrial Respiration in Diabetic Patients Treated with Statins

**Figure 4:**
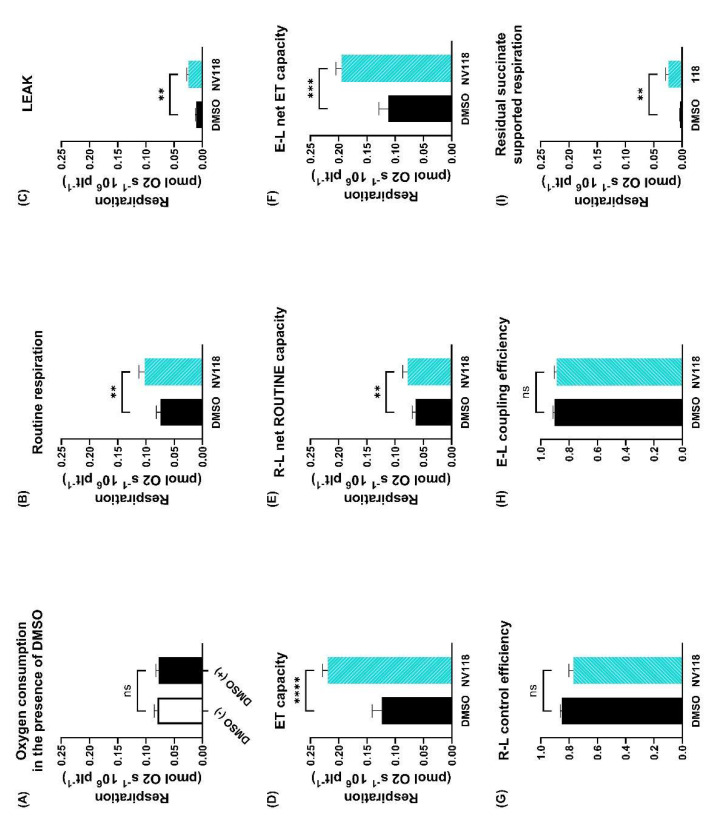
**Platelet mitochondrial bioenergetics of diabetic patients treated with statins in the presence vs. absence of NV118.** To confirm that the addition of DMSO (1 µL) does not have an effect per se on ROUTINE respiration, oxygen consumption was assessed in the presence vs. the absence of DMSO (**A**). ROUTINE respiration (**B**), LEAK (**C**), ET capacity (**D**), R–L net ROUTINE capacity (**E**), E–L net ET capacity (**F**), R–L control efficiency (**G**) and E–L coupling efficiency (*H*) were determined for the statin–treated diabetic patients in the presence of the cell–permeable succinate NV118 (cyan columns) vs. the absence (black columns). Residual succinate–supported respiration (oxygen consumption determined after the addition of rotenone, a potent inhibitor of complex I) (*I*). N = 8. Data are expressed as the mean ± SEM of antimycin A–corrected respiration. Paired *t*–tests were performed. ns = no statistical significance vs. control (except for A where it ns = no statistical significance between the presence/absence of DMSO); ** *p* < 0.01; *** *p* < 0.001; **** *p* < 0.0001 vs. control. DMSO = dimethyl sulfoxide; E = electron transfer capacity; ET capacity = electron transfer capacity; L = LEAK respiration; P = oxidative phosphorylation; R = ROUTINE respiration.

In order to assess whether NV118 can improve mitochondrial bioenergetics, we determined the parameters of mitochondrial respiration for statin–treated diabetic patients in platelets incubated with NV118 and an equivalent amount of DMSO (its solvent). The addition of DMSO (1 µL), used as the control for the experiments with NV118, did not modify platelet respiration, as shown in Figure 4A (0.079 ± 0.005 vs. 0.077 ± 0.005). In the presence of NV118, ROUTINE respiration (Figure 4B) increased from 0.074 ± 0.007 to 0.102 ± 0.010 (*p* < 0.01), LEAK respiration (Figure 4C) increased from 0.011 ± 0.001 to 0.024 ± 0.003 (*p* < 0.01), as did ET capacity (Figure 4D) from 0.123 ± 0.017 to 0.219 ± 0.009 (*p* < 0.0001), respectively. To measure ATP–generating respiration, R–L net ROUTINE capacity (Figure 4E) was calculated as the difference between ROUTINE respiration and LEAK respiration, which showed a slight increase from 0.063 ± 0.006 to 0.078 ± 0.008 (*p* < 0.01). To determine the maximal potential respiration available for ATP–generation, E–L net ET capacity (Figure 4F) was calculated as the difference between ET capacity and LEAK respiration, which showed an increase from 0.112 ± 0.016 to 0.194 ± 0.009 (*p* < 0.001). As a measure of the degree of coupling, we calculated the R–L control efficiency (Figure 4G, 0.851 ± 0.012 vs. 0.768 ± 0.032) and E–L coupling efficiency (Figure 4H, 0.902 ± 0.013 vs. 0.888 ± 0.017), respectively. No statistical significance was found between control samples and those exposed to NV118. These data indirectly suggest that mitochondrial oxygen consumption generates more ATP in the presence of NV118, as oxygen consumption is increased while the efficiency of the ETS remains largely unaltered. In order to assess whether the cell–permeable succinate was properly delivered, oxygen consumption was measured after the addition of rotenone (a potent inhibitor of complex I), thus resulting in residual succinate–supported respiration that is significantly increased in the presence of NV118 (Figure 4I).

## 4. Discussion

The main findings of the study are as follows: (i) in chronic administration statins are safe for platelet mitochondrial respiration and (ii) cell–permeable succinate is capable of improving mitochondrial respiration in diabetic patients treated with statins. Our data are in line with the observations by Vevera et al., who also used isolated platelets to evaluate mitochondrial function in patients treated with simvastatin and found no significant effect on mitochondrial respiration, suggesting the occurrence of in vivo compensation [29]. Interestingly, the group of Rasmussen [30] recently reported a higher mitochondrial oxygen consumption (increase in complex I activity) in simvastatin–treated patients. These findings further add to the surmounting proof of the past four decades that statins are safe under normal therapeutic conditions [2]. In another study with statin–treated patients, Gvozdjakova et al. [31] showed that atorvastatin and fluvastatin improved platelet mitochondrial respiration. The mitochondrial toxicity induced by statins has been firstly reported by Kaufman et al. [17], who showed the dissipation of mitochondrial membrane potential in the presence of cerivastatin, atorvastatin, fluvastatin and simvastatin and decreased glutamate–driven state 3 (ATP–generating) respiration for all mentioned statins (but fluvastatin). However, while skeletal muscle mitochondrial dysfunction could be demonstrated for a statin dose of 100 µmol/L [17], the same result could not be recapitulated by other authors that used a much lower dose (5 µmol/L) [32].

The most plausible explanation for the common culpability of statins to induce acute organ–related mitochondrial dysfunction while not doing the same in experiments assessing the effects of chronic treatment is their dose dependency [33]. Of note, both atorvastatin and rosuvastatin have maximum therapeutic concentrations in the nanomolar range in human plasma [34,35]. Whether high concentrations of statins can be reached in vivo is a matter of debate. 

The most severe (yet fortunately rare) side effect of statin therapy is rhabdomyolysis, and the factors that favor this dreaded side effect are: treated diabetes, hypothyroidism, vitamin D deficiency, genetics, age, gender, ethnicity, statin dose, alcohol intake and concomitant medication [10,13,36,37,38,39]. Because statins are metabolized through the cytochrome P450 pathway, inhibitors of this pathway may lead to statin accumulation and increased plasma levels [40]. Statin treatment can easily be associated in practice with medications that inhibit the cytochrome P450 pathway such as: amiodarone, gemfibrozil, verapamil, diltiazem, cyclosporine, azole antifungals, macrolide antibiotics or HIV protease inhibitors [1,37,41,42,43,44]. Diabetes has also been associated with a reduction in the expression of genes that encode components of the mitochondrial respiratory chain, among which two were attributed to complex I, thus interfering with mitochondrial OXPHOS [45]. 

Mitochondrial dysfunction and reduced mitochondrial content are widely acknowledged pathomechanisms of type 2 diabetes [46,47]. Experiments carried out in permeabilized muscle cells have shown a reduced mitochondrial oxygen consumption in diabetic patients [48,49,50]. Similar results have been found by Avila et al. using platelets from diabetic patients [51]. However, other groups showed that no differences in mitochondrial respiration in the setting of diabetes were found when the results were normalized to the mitochondrial content [48,49]. 

Statins have been reported to cause uncoupling in rat myoblasts as shown by the pioneering work by Kaufmann et al. [17]. More recently, Broniarek et al. reported that atorvastatin (and, in some cases, pravastatin) causes uncoupling (increased LEAK respiration) in endothelial cells [52]. LEAK respiration is a dissipative component of mitochondrial respiration that is nonphosphorylating and determines heat production [26,27]. In our study, we did not find a significantly increased LEAK respiration between the statin–treated groups and the untreated controls in the experiments carried out in intact platelets. We speculate that the statin level in these patients was much lower as compared with the doses used in the majority of the ex vivo studies (up to 1000–fold higher than the plasma concentration of statins under therapeutic conditions) [17,34,35,52,53]. 

Accordingly, as depicted in Figure 2G,F, mitochondrial respiration is just as efficient with statin treatment as it is without it. As the values of the E–L coupling efficiency and that of the R–L control efficiency are close to 1.0, it means that the ETS is close to a fully coupled (ATP–generating) system [27]. 

DMSO was used as a control for the experiments with NV118 as it is the most common universal solvent. The dose of DMSO (1 µL) that was used was very small and did not affect mitochondrial respiration, as shown in Figure 4A. DMSO can indeed be toxic but in higher doses (above 1%) [24]. 

Lower mitochondrial phosphorylation has been reported to play a key role in insulin resistance and the pathophysiology of type 2 diabetes [54,55]. Thus, Sadighara et al. [56] showed that high concentrations of atorvastatin (above 75 µM) decreased ATP levels in pancreatic cells from Sprague–Dawley rats. It has been postulated that impaired ATP production will lower insulin secretion rates [8]. 

Statins are classically associated with the increased risk of developing type 2 diabetes in particular patients [14]. Despite the fact that statins have been reported to increase LEAK respiration [17,23], we showed here that atorvastatin did not elicit such an effect when chronically administered in diabetic patients (Figure 2B and Figure 3C). It is tempting to speculate that the statin–induced increase in LEAK respiration may be responsible for new–onset diabetes [8,14,56] because new–onset diabetes occurred more frequently in elderly patients on high–dose statin therapy [14]. Old age itself determines enzymatic changes, leading to higher LEAK, lower OXPHOS and ATP generation [57,58], while statin–induced mitochondrial dysfunction has clearly been shown to be concentration dependent [17,23]. Increasing OXPHOS to compensate for LEAK can maintain P–L control efficiency and E–L coupling efficiency (i.e., ATP production) [59]; therefore, cell–permeable succinates, such as NV118, may be prime candidates to achieve this. Even though statins are known to be responsible for the onset of new diabetes, the benefits in reducing coronary events far outweigh the cost, making statin treatment mandatory to reduce cardiovascular risk [1,14,60].

Because diabetes and statin treatments are a common association and each can inhibit mitochondrial complex I, there is an unmet need for a treatment that bypasses this inhibition altogether [1,5,15,16,17,18,19]. Cell permeable succinates are novel agents that can improve mitochondrial function in the settings of impaired NADH–linked respiration, including very high concentrations of statins [21,22,23,61]. 

We have found that in the presence of a cell–permeable succinate ROUTINE respiration, R–L net ROUTINE capacity, ET capacity and also, E–L net ET capacity can be increased in diabetic patients treated with statins. The fact that there is no statistical difference between P–L control efficiency and E–L coupling efficiency of the NV118–treated platelets vs. control (DMSO) suggests that the level of uncoupling is the same [27,59,62]. This could mean that ATP is being generated in higher amounts by the NV118–treated platelets as the oxygen consumption increases while preserving the degree of coupling. Although ATP production depends on mitochondrial oxygen consumption, it also depends on the substrate being oxidized [63,64]. For succinate (complex II substrate), the H^+^/O ratio is 6, while malate and pyruvate (complex I substrates) present a ratio of 10 [63]. We acknowledge as an important limitation of the present study the fact that the platelet ATP level was not assessed.

The use of statins has unequivocal benefits in cardiometabolic pathologies in both young and elderly patients [1,2,60,65]. Due to their pleiotropic effects, statins are useful not only in treating dyslipidemia per se but also concomitant risk factors such as hypertension [66], further contributing to the reduction of cardiovascular risk. Recent reports indicate that statins also improve the outcome in inflammatory diseases such as psoriasis, especially in severe cases [67]. 

Among their pleiotropic effects, statins have been reported to interfere with the redox signaling in platelets by inhibiting the NADPH oxidase–derived ROS formation [68,69]. Increased mitochondrial ROS generation has been systematically reported as a central pathomechanism associated with hyperglycemic conditions and metabolic syndrome leading to platelet activation; in turn, platelet–derived ROS trigger an auto–amplifying loop of persistent ROS production and sustained platelet activation that underlie the increased thrombotic risk in diabetes [70,71,72]. Beyond the stabilizing effects of atheroma plaques, statins inhibit platelet activation and have antioxidant properties and antithrombotic effects [69,71,73].

We still face currently the fact that elderly patients (often at higher cardiovascular risk) do not take adequate doses of lipid–lowering drugs and do not reach their intended treatment targets [65,74]. Moreover, the poor statin adherence of patients suffering from statin–induced muscle symptoms is a clinical reality [75]. Albeit mitochondrial dysfunction may occur in the skeletal muscle, chronic administration of statins in diabetic patients does not cause a decrease in mitochondrial respiration in peripheral platelets. Further investigation is warranted to assess whether changes in platelet respiration can mirror the response of skeletal muscle to high–dose statin treatment [29,30,76].

## 5. Conclusions

Statins remain the cornerstone of both the treatment and prevention of cardiometabolic pathologies. Because there are certain factors that predispose to statin–induced side effects, there is an unmet need for supporting mitochondrial function, particularly in aging patients with comorbidities. Cell–permeable succinates have recently emerged as viable candidates able to increase mitochondrial function in several pathologies. It is tempting to speculate that the use of cell–permeable succinates could increase statin adherence by improving mitochondrial bioenergetics and, possibly, reducing the side–effects. Nevertheless, multicenter intervention studies are required in order to confirm these findings and the usefulness of the cell–permeable succinate compounds for clinical care.

## Data Availability

Data are contained within the article.

## Supplementary Materials

The following are available online at https://www.mdpi.com/article/10.3390/life11040288/s1, Table S1: Characteristics of study participants, Table S2: Comorbidities and concomitant medication of study participants.
