# Supplementary material for: Improvement of Platelet Respiration by Cell–Permeable Succinate in Diabetic Patients Treated with Statins
Source: Life (Basel). 2021 Mar 28;11(4):288. doi: 10.3390/life11040288 (PMC8065590; doi:10.3390/life11040288)
Supplement: Supplementary file 1 [file life-11-00288-s001.pdf]

Table S1. Characteristics of study participants.

No.	Age	Gender	TC (mg/dL)	LDL-C (mg/dL)	TG (mg/dL)	HDL-C (mg/dL)	LDL-C/HDL-C	Thrombotic events	Statin dosage	Duration of diabetes (y)
1	47	F	253	155	271	47	3.30	NO	-	6
2	68	F	244	156	220	57	2.74	NO	-	15
3	39	M	215	154	148	31	4.97	NO	-	2
4	66	F	186	116	183	33	3.52	YES	-	12
5	63	M	171	119	95	54	2.20	YES	Atorvastatin 10 mg	20
6	60	F	149	73	154	49	1.49	NO	Atorvastatin 10 mg	11
7	79	M	113	67	141	30	2.23	NO	Atorvastatin 20 mg	10
8	69	F	194	106	96	69	1.54	NO	Atorvastatin 20 mg	5
9	58	M	132	85	108	34	2.50	YES	Atorvastatin 40 mg	14
10	62	M	152	79	280	37	2.14	YES	Rosuvastatin 10 mg	15
11	67	M	164	109	173	30	3.63	NO	Rosuvastatin 10 mg	14
12	53	M	119	60	42	47	1.28	NO	Rosuvastatin 10 mg	6
13	48	F	143	83	200	32	2.59	NO	Rosuvastatin 20 mg	19

Table S2. Comorbidities and concomitant medication of study participants.

No.	Hypertension	Retinopathy	Chronic kidney disease	Cerebrovascular disease	Coronary heart disease	Antiplatelet medication	Antihypertensive medication
1	YES	YES	NO	NO	YES	NO	perindopril; indapamid; nebivolol
2	YES	NO	NO	NO	YES	NO	candesartan; amlodipin;
3	YES	NO	NO	NO	NO	NO	perindopril
4	YES	YES	NO	NO	NO	YES	perindopril; indapamid; nebivolol
5	YES	YES	YES	YES	YES	YES	perindopril; amlodipin; bisoprolol
6	YES	NO	NO	NO	NO	YES	perindopril; indapamid; nebivolol
7	YES	NO	NO	YES	NO	NO	ramipril; furosemide; spironolactone; bisoprolol
8	YES	NO	NO	NO	NO	NO	perindopril
9	YES	YES	YES	NO	YES	YES	perindopril; indapamid; nebivolol; amlodipin
10	YES	NO	YES	YES	YES	NO	perindopril; indapamid; nebivolol; amlodipin
11	YES	NO	YES	NO	NO	YES	perindopril; indapamid; carvedilol; amlodipin
12	YES	NO	NO	NO	NO	NO	perindopril; indapamid
13	YES	NO	YES	NO	NO	NO	candesartan; nebivolol
